# Combined flow cytometry natural killer immunophenotyping and KIR/HLA-C genotyping reveal remarkable differences in acute myeloid leukemia patients, but suggest an overall impairment of the natural killer response

**DOI:** 10.3389/fmed.2023.1148748

**Published:** 2023-03-07

**Authors:** Vlad Andrei Cianga, Cristina Rusu, Mariana Pavel-Tanasa, Angela Dascalescu, Catalin Danaila, Sebastian Harnau, Carmen-Mariana Aanei, Petru Cianga

**Affiliations:** ^1^Department of Hematology, University of Medicine and Pharmacy “Grigore T. Popa”, Iasi, Romania; ^2^Department of Clinical Hematology, Regional Institute of Oncology, Iasi, Romania; ^3^Department of Genetics, University of Medicine and Pharmacy “Grigore T. Popa”, Iasi, Romania; ^4^Department of Immunology, University of Medicine and Pharmacy “Grigore T. Popa”, Iasi, Romania; ^5^Laboratory of Hematology, Nord Hospital, CHU Saint Etienne, Cedex2, Saint-Étienne, France; ^6^INSERM U1059-SAINBIOSE, Université de Lyon, Saint-Étienne, France

**Keywords:** NK, AML, KIR, NKG2A, HLA-C, FLT3, lymphocytes

## Abstract

**Introduction:**

Natural killer (NK) cells are key anti-tumor effectors of the innate immunity. Phenotypic differences allow us to discriminate in between three functional stages of maturation, named immature, mature and hypermature that are distinctive in terms of receptor expression, cytokine secretion, cytotoxic properties and organ trafficking. NKs display an impressive repertoire of highly polymorphic germline encoded receptors that can be either activating, triggering the effector’s function, or inhibitory, limiting the immune response. In our study, we have investigated peripheral blood NK cells of acute myeloid leukemia (AML) patients.

**Methods:**

The Killer Immunoglobulin-like receptors (KIRs) and the HLA-C genotypes were assessed, as HLA-C molecules are cognate antigens for inhibitory KIRs.

**Results:**

The AA mainly inhibitory KIR haplotype was found in a higher proportion in AML, while a striking low frequency of the 2DS3 characterized the mainly activating Bx haplotype. Flow cytometry immunophenotyping evidenced a lower overall count of NK cells in AML versus healthy controls, with lower percentages of the immature and mature subpopulations, but with a markedly increase of the hypermature NKs. The analysis of the KIR2DL1, KIR2DL2, KIR2DL3, KIR3DL1, and NKG2A inhibitory receptors surface expression revealed a remarkable heterogeneity. However, an overall trend for a higher expression in AML patients could be noticed in all maturation subpopulations. Some of the AML patients with complex karyotypes or displaying a FLT3 gene mutation proved to be extreme outliers in terms of NK cells percentages or inhibitory receptors expression.

**Discussion:**

We conclude that while the genetic background investigation in AML offers important pieces of information regarding susceptibility to disease or prognosis, it is flow cytometry that is able to offer details of finesse in terms of NK numbers and phenotypes, necessary for an adequate individual evaluation of these patients.

## 1. Introduction

Acute myeloid leukemias (AML) are particularly heterogeneous hematological cell malignancies characterized by myeloid cells maturation blockade, intense proliferation of hematopoietic precursors and bone marrow insufficiency (1). The prognosis is usually poor, and in terms of clonality, phenotype and cytogenetic alterations, there are well defined entities established by the World Health Organization (WHO) (2) and the European Leukemia Network (ELN) (3). The unfavorable prognosis reflects not only the challenges faced by the immune system, but also by the various therapeutic approaches (4–6).

The natural killer cells, initially characterized in the context of their ability to nonspecifically kill mouse tumor cells (7) are now acknowledged as members of the Innate Lymphoid Cells (ILC) capable of killing not only tumor cells, but also virally infected and damaged cells, acting together with the T cytotoxic cells within the “Cytotoxicity” immune effector module (8, 9). Furthermore, NK cells were shown to secrete a number of cytokine that are involved in regulating/altering the activity of innate as well as the specific immunity (9, 10).

Even though they derive from the same common lymphoid progenitor (CLP), unlike T cells, NK cells lack genetically rearranged specific antigen receptors (10). However, they express an impressive array of germline encoded receptors that are remarkably polymorphic. Although multiple receptor classes are currently acknowledged, an important distinction between them is based on their activating or inhibitory properties. The activating receptors, such as those belonging to the natural cytotoxic receptors (NCR) family (ex. NKp30, NKp46), and the activating Killer Immunoglobulin-like Receptors (aKIRs) are stimulated by ligands that signal cellular damage, viral infection or tumor transformation (11, 12). On the other hand, the inhibitory receptors represented by the CD94/NKG2A heterodimer and the inhibitory KIRs (iKIRs), recognize self HLA class I molecules (both classical and non-classical) on cells that will thus evade NK mediated destruction (12, 13).

The classical HLA-C molecules are such ligands that are able to interact with the majority of iKIRs. On the basis of an asparagine vs. lysine dimorphism at position 80 of the α1 domain, the HLA-C molecules fall in two groups named C1 (HLA-C*01/*03/*07/*08/*12/*14/*16) and C2 (HLA-C*02/*04/*05/*06/*15/*17/*18) that offer two major KIR epitopes (14, 15).

To date, 17 KIR genes are known, six of them encoding the KIR2DS2, KIR2DS3/2DS5C, KIR3DS1, KIR2DS3/2DS5T, KIR2DS4, and KIR2DS1 activating receptors, nine of them encoding the KIR3DL3, KIR2DL2, KIR2DL3, KIR2DL5B, KIR2DL1, KIR2DL4, KIR3DL1, KIR2DL5A, and KIR3DL1 inhibitory receptors, while KIR2DP1 and KIR3DP1 are pseudogenes, with yet undefined functions (16, 17). Based on the various combinations of KIR genes (including pseudogenes), several KIR genotypes could be characterized. Further, based on these genotypes, two major haplotypes, named AA and Bx, could be defined. The AA haplotype includes the activating KIR2DS4 gene and the inhibitory KIR2DL1, KIR2DL3, KIR3DL1 genes, while the Bx haplotype includes all the other KIR genotypes (16). Due to deletions that render the KIR2DS4 gene non-functional, about 70% of Caucasians that are homozygous for the AA haplotype do not express this receptor (18). It is thus generally acknowledged that AA and Bx haplotypes, due to the predominance of iKIRs over aKIRs, confer distinctive NK cells functions (19).

KIR2DL1 (CD158a) and KIR2DL2/2DL3 (CD158b) bind to HLA-C, while KIR3DL1 (CD158e1) binds to HLA-A and HLA-B. The absence of such molecules on the surface of a particular cell (generally described as “missing self”) makes it a target for the NK attack. KIR2DL1 bind to HLA-C2, while KIR2DL2 and KIR2DL3 bind to HLA-C1. KIR3DL1 binds to the Bw4 epitope of the HLA-A and B molecules (20). NKG2A (CD159a) pairs with CD94 to make an inhibitory receptor that binds to HLA-E, a non-classical MHC (21).

Various studies have shown that in pathological settings, NK cells display an altered profile, attributed to the direct action of the malignant cells. The resistance of leukemic cells against NK cells occurs both in the peripheral blood (PB) compartment, as well as in the modified microenvironment of the bone marrow (BM) (22, 23). Thus, anti-leukemic NK cells are often reduced in numbers and they present a reduced receptor expression while their cognate ligand is also downregulated (24–30).

The NK cells ability to interact with self HLA molecules is gained in a process called NK “education” (13) and it is critical for the functional maturity of the cell. Functional NK cells can be separated in 3 subpopulations, based on their level of maturity and role. Firstly, the more immature CD56^bright^CD94 + CD16-NKG2A + KIR- NK cells have been associated with cytokine and soluble factors secretion (tumor necrosis factor α, TNFα; interferon γ; IFNγ) which support inflammation, macrophage recruitment and activation of lymphocytes and dendritic cells (31, 32). These NK cells evolve further into the mature CD56^dim^CD94+/-CD16 + NKG2A + KIR+ subpopulation, which is involved in cytotoxic cell lysis mediated by granzyme B and perforin release and also by antibody dependent cytotoxicity (ADCC) (33–35). The mature subpopulation CD56^dim^CD16 + KIR+ subpopulation can further gain CD57, a marker associated with higher cytotoxic potential, superior stimulation through CD16, decreased proliferative capabilities and even long-term cellular memory (36, 37).

This is a more in-depth follow-up study of a previous NK cells analysis in the context of myelodysplastic syndromes and AMLs (38). In the present study, we focused exclusively on AML patients. We evaluated both the NK cells KIR genotypes and phenotypes to better discriminate among NK subpopulations and to potentially assess in which way the NK anti-tumor response is hindered in this particular malignancy.

## 2. Materials and methods

### 2.1. Patients and controls

This study included 20 newly diagnosed AML patients. They were admitted and diagnosed in the Regional Institute of Oncology (Iasi, Romania) in a time frame of 6 months (May – October 2022). The characteristics of the subjects are presented in Table 1. AML diagnosis and risk stratification were made based on the European Leukemia Network 2022 (ELN) criteria (3). Table 2 summarizes the recurrent genetic abnormalities, the normal and the complex karyotypes and the distribution of the patients within the favorable (*n* = 2), intermediate (*n* = 10) and unfavorable (*n* = 8) groups.

**Table 1:** Subjects characteristics.

Characteristics	AML group	Healthy control
Subjects	20	20
AGE MEAN ± SEM	60.74 ± 3.36	64.87 ± 5.27
Male/female ratio	12/7 63.1%/36.9%	12/8 60%/40%
Hemoglobin mean ± SEM	8.58 ± 0.47 g/dL	13.7 ± 0.19 g/dL
Platelets mean ± SEM	117 ± 22.6 × 10^9^/L	225 ± 16.2 × 10^9^/L
Leucocytes mean ± SEM	6.48 ± 1.76 × 10^9^/L	9.75 ± 02.18 × 10^9^/L
Lymphocytes mean ± SEM	1.68 ± 0.38 × 10^9^/L	1.63 ± 0.39 × 10^9^/L
Bone marrow blasts (%) mean ± SEM	41.7 ± 4.5 (%)	–

AML, acute myeloblastic leukemia; SEM, standard error of mean.

**Table 2:** Risk stratification of AML cases based on their karyotype and recurrent molecular abnormalities.

Karyotype	Recurrent molecular abnormalities	ELN risk stratification
46XX/46XY (*n* = 13)	FLT3-ITD (*n* = 1)	Favorable (*n* = 2)
Complex karyotype (*n* = 7)	NPM1 (*n* = 2)	Intermediate (*n* = 10)
		Unfavorable (*n* = 8)

ELN, European leukemia network.

A group of 20 age matched volunteers was enrolled for the phenotype control analyses, while for the KIR and HLA-C genotype analyses we used a group of 98 healthy volunteers that were selected from the Romanian National Registry of Hematopoietic Stem Cell Donors. The study was approved by the Institutional Ethics Committee and consent was obtained from each patient (343/15.11.2018).

### 2.2. Flow cytometry

#### 2.2.1. Sample preparation and staining

A total of 400 μL of peripheral blood (PB) were collected in EDTA tubes for each patient, and further aliquoted equally in 4 tubes. One sample had to be excluded from the analysis due to clotting issues. The sample preparation procedures and the staining strategy were previously described in detail (38, 39). The monoclonal antibodies we have used are summarized in Supplementary Table S1. Briefly, cells in each tube were stained with monoclonal antibodies aimed to define the NK subpopulations and thereafter each such tube was stained with a monoclonal antibody targeting one of the following NK receptors: CD158a (KIR2DL1), CD158b (KIR2DL2/2DL3), CD158e1 (KIR3DL1) and CD159a (NKG2a). This approach was imposed due to fluorochrome-related constraints hence it made possible the characterization of just one receptor at a time, which limited us in evaluating the cells expressing multiple types of inhibitory NK receptors.

A total of 500.000 events per/sample were acquired on a FACSCanto II cytometer (BD Biosciences, San Jose, CA, United States) with the FACSDiva v1.6 acquisition software.

#### 2.2.2. Data analysis

Data were analyzed with the Cytognos Infinicyt v2.0 software. NK cells were gated according to the CD45+/CD56+/CD3-/CD19- pattern of expression that excluded T (CD45 + CD3 + CD19-CD56-) and B lymphocytes (CD45 + CD19 + CD3-CD56-). NK subpopulations were identified based on the expression patterns combination of the CD56, CD94, CD16, and CD57 markers. The immature subpopulation was defined as CD56^bright^CD94^hi^CD16 − CD57−, the mature subpopulation as CD56^dim^CD94^med^CD16 + CD57−, and the hypermature subpopulation as CD56^dim^CD94lowCD16 + CD57+. For analyzing the flow cytometry data in a reproducible and objective manner we took advantage of the Principal Component Analysis (PCA), an algorithm implemented to reduce data variance and interpret multidimensional information (40). Infinicyt’s™ automatic population separator (APS) tool uses PCA to compress information in bidimensional plots. This method helped us classify NK cells in the above mentioned three maturation subsets.

The KIR and NKG2A receptors (CD158a, CD158b, CD158e1, and CD159a) were evaluated in each NK cell subpopulation of the AML and control groups.

The mean fluorescence intensity (MFI) of the NK markers was analyzed in all subpopulations using box-plots. The gating strategy for identification of the NK cell subpopulations and the KIR receptors is summarized in Supplementary Figure S1.

Regarding the intensity of the CD158a, CD158b, CD158e1 and CD159a receptors expression, the mean fluorescence intensity (MFI) was evaluated. The acquisition of NK cells was performed on two identical cytometers, with the same calibration and experimental parameter setup; 8-peak Rainbow beads were used in both cytometers as quality control. However, in order to eliminate potentially false results and harmonize the data, we calculated the MFI index (mean of receptor positive NK population/mean of receptor negative NK positive population).

### 2.3. Killer Immunoglobulin-like receptor and HLA-C genotyping by polymerase chain reaction – sequence specific primers

#### 2.3.1. Killer Immunoglobulin-like receptor genotyping

PB harvested on EDTA was subjected to DNA extraction using either the silica adsorption columns based method (QIAamp DNA Blood Mini Kit, Qiagen, Germany) or an automatic method reliant on magnetic separation (AB Library Builder, Life Technology, United States).

The KIR genotypes were assessed for all 20 AMl patients and 98 healthy controls using commercially available PCR-SSP (polymerase chain reaction – sequence specific primers) kits from the following manufacturers: Inno Train (Inno Train Diagnostik GmbH, Kronberg, Germany), Invitrogen (ThermoFisher Scientific Inc., Waltham, United States), Olerup (Olerup, Stockholm, Sweden) and BAG Diagnostics (BAG Diagnostics, Lich, Germany). KIR patterns were interpreted using the Specificity Tables available for each such commercial kits. However, since these different kits amplify slightly different allelic variants of the investigated genes, we interpreted the data at low resolution in order to pool the final results.

#### 2.3.2. HLA-C genotyping

The HLA-C genotyping could only be performed for 15 AMl patients due to a number of technical issues and on all 98 healthy controls used for KIR genotyping. A similar PCR-SSP approach was used, based on the Olerup (Olerup, Stockholm, Sweden) commercially available kits. The amplicons pattern was analyzed with the Score software.

### 2.4. Statistical analysis

We performed the statistical analysis using Graph Pad Prism 8.0.1™ (Graph Pad Software, San Diego, CA, United States) and SPSS v20™ (IBM SPSS Software, Chicago, IL, United States). Figures and tables were created in Graph Pad Prism 8.0.1™. Bar graphs and scatter dot plots show means with standard errors of means (SEM). We analyzed the differences in means of normally distributed variables using the unpaired *t*-test, and of non-Gaussian distributed data using the Mann–Whitney non-parametric test. Distribution of each KIR gene in the AML and healthy control groups was determined by the use of contingency tables and Chi squared test was calculated to determine the significance of the differences between various allelic groups. One-sample t-tests were used for analyzing the individual ratios of various NK cell populations and Pearson’s and Spearman’s correlation coefficients were implemented to assess positive associations between the measured variables. The *p*-value below 0.05 was considered significant, and R higher than 0.5 or lower than −0.5 was considered a strong correlation factor.

## 3. Results

### 3.1. Natural killer subpopulations in acute myeloid leukemia settings

**Figure 1:**
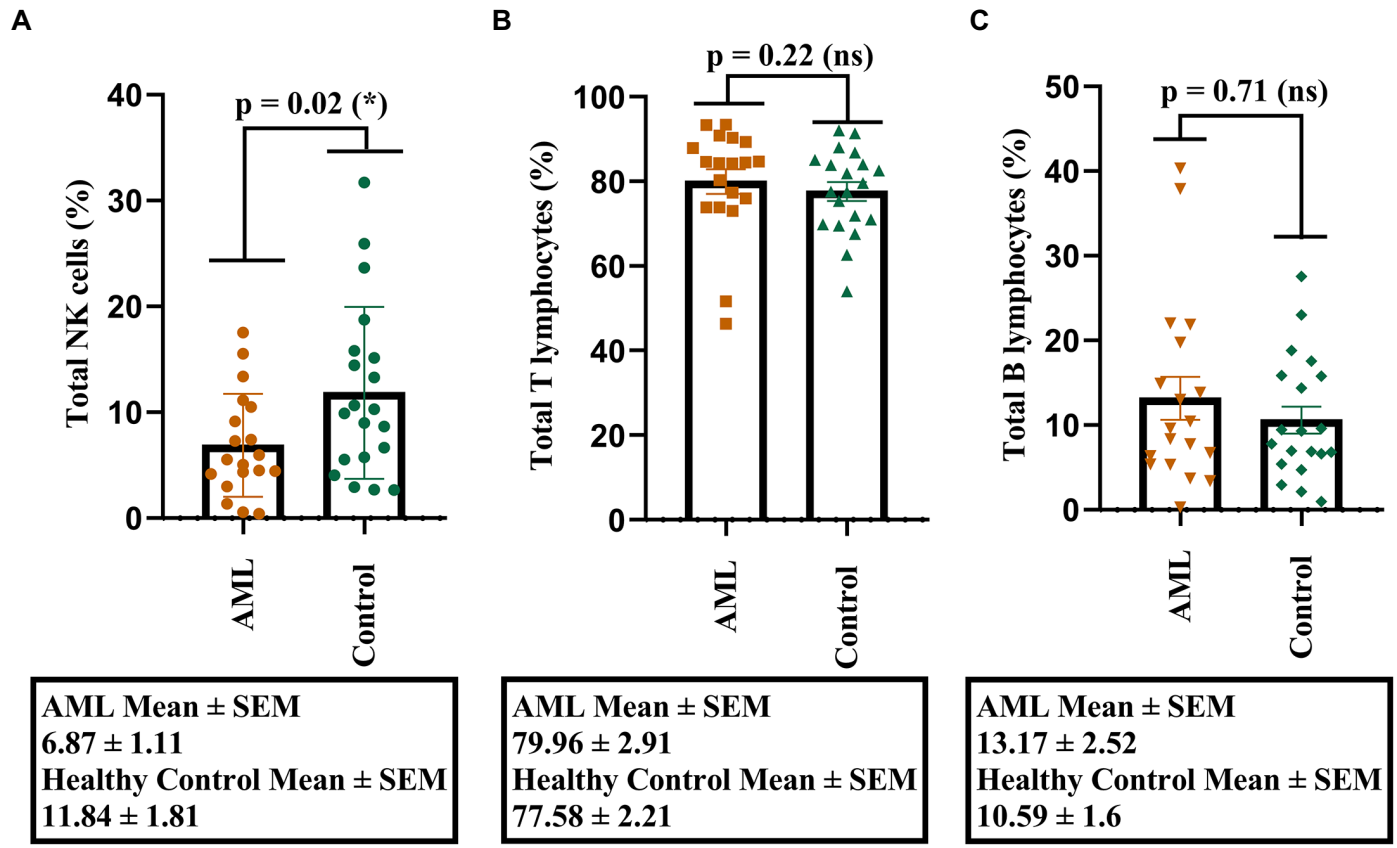
Percentage of lymphocytes in AML and healthy controls. **(A)** Comparison between the NK cell percentages (%) in AML (*n* = 19) and healthy control (*n* = 20) cases. **(B)** Comparison between the T cell percentages (%) in AML (*n* = 19) and healthy control (*n* = 20) cases. **(C)** Comparison between the B cell percentages (%) in AML (*n* = 19) and healthy control (*n* = 20) cases. Bars represent the mean ± SEM (*p* < 0.05 (*); ns, not significant; two tailed unpaired *t*-tests).

Our first objective was to investigate the percentages of total NK, T, and B cells and to identify, if any, the potential differences. A lower percentage of NK cells was found in the AML group compared to the control group (*p* = 0.02; Figure 1A), but no significant differences were noticed for the T and B cell populations (Figures 1B,C). Another observation is that the lowest percentages of NK cells belonged to a case with FLT3 mutation (0.53% NK cells) and to one with complex karyotype (0.38% NK cells).

**Figure 2:**
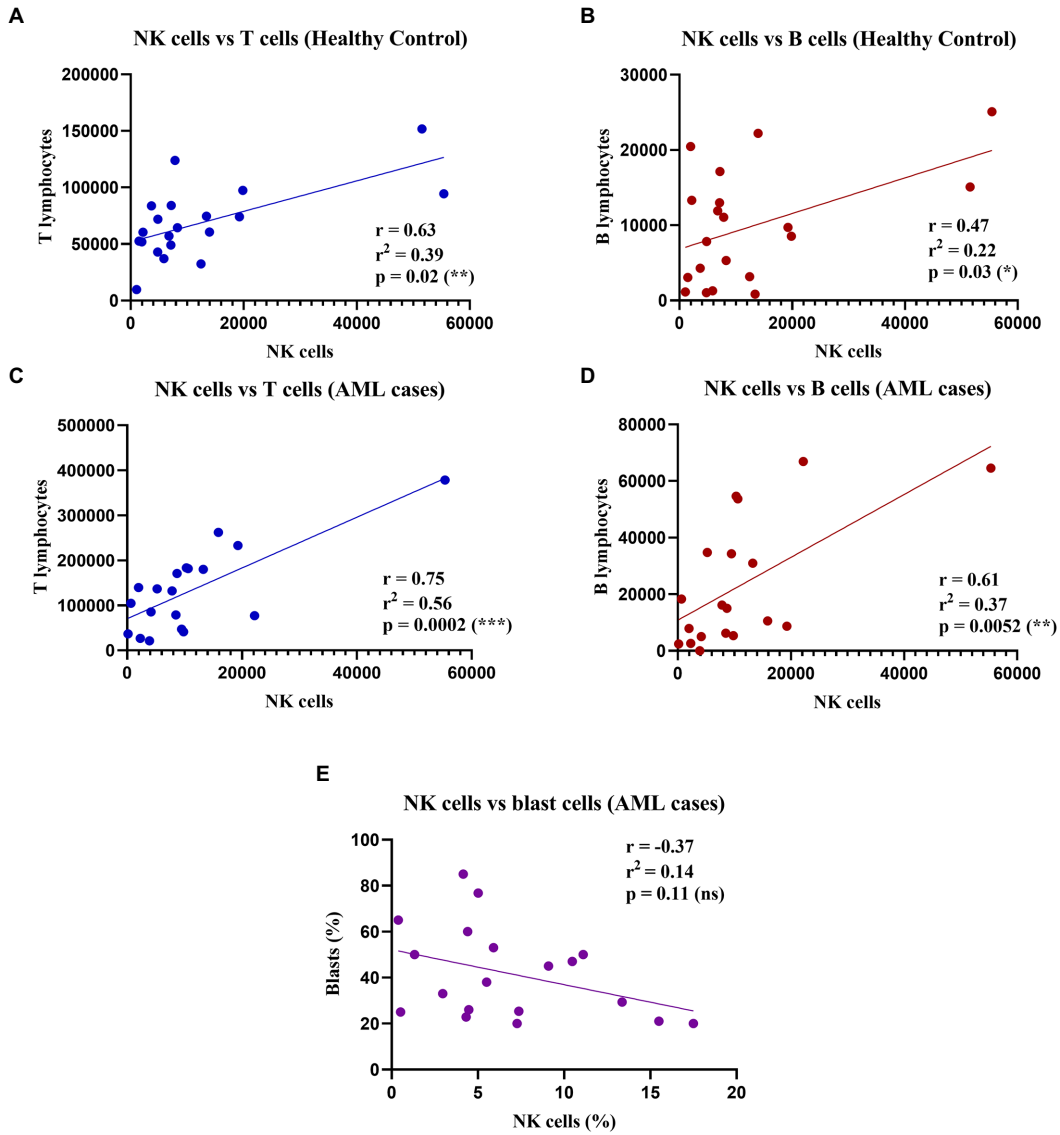
Correlations between NK, T, and B cell counts in healthy control and AML cases. (Top) Linear regressions for the healthy control group: **(A)** Between NK cells and T cells (*n* = 20); *p* = 0.02 (*). **(B)** Between NK cells and B cells (*n* = 20); *p* = 0.03 (*). Bottom linear regression for the AML group: **(C)** Between NK cells and T cells (*n* = 19); *p* = 0.0002 (***). **(D)** Between NK cells and B cells (*n* = 19; *p* = 0.005 (**). **(E)** Between percentages (%) of NK cells and (%) blast cells (*n* = 19; ns, not significant).

As the percentage difference was only observed for the NK cells, we wondered whether this decrease was accompanied by any changes in the total number of the other T and B lymphocytes. Thus, we assessed any potential positive or negative correlations between the total number of NK cells and the other lymphocyte types (T and B cells), as well as with the number of blast cells that were investigated in the bone marrow aspirate harvested for the AML diagnosis (Figure 2).

Our results showed significant moderate positive correlations between the number of NK, and T and B lymphocytes respectively, in both the control and the AML groups (Figures 2A–D). Furthermore, we sought to evaluate if there is a correlation between the leukemic blasts and the percentage of NK cells in peripheral blood, but no correlation was found between these populations (r = −0.37; *p* = 0.11; Figure 2E).

**Figure 3:**
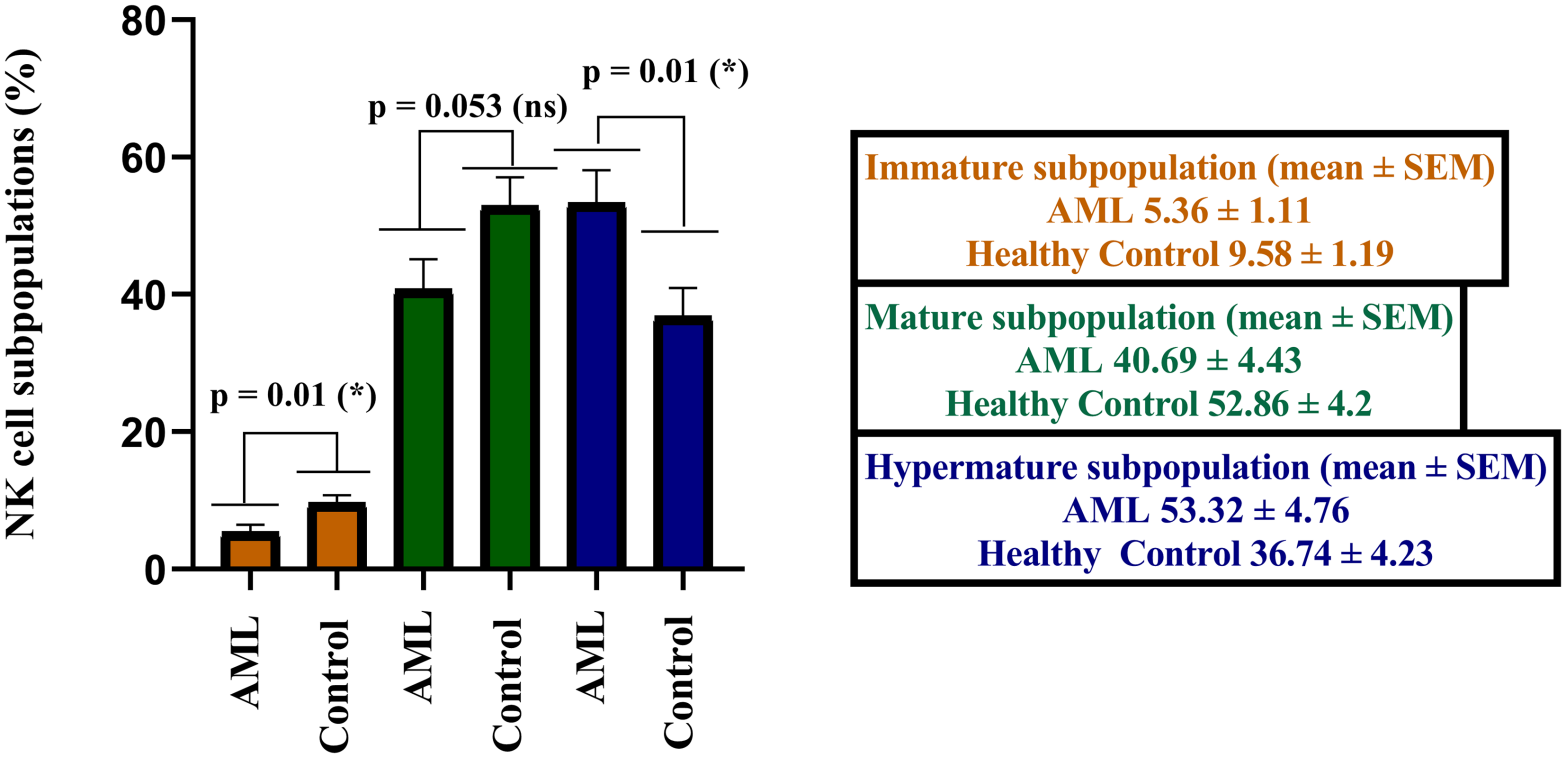
Comparison of cell percentages in distinct NK maturation subpopulations of AML (*n* = 19) and healthy control (*n* = 20) – immature (orange), mature (green) and hypermature (blue). Bars represent the mean ± SEM (p < 0.05 (*); ns, not significant; two tailed unpaired *t*-tests).

When analyzing the immature, mature and hypermature NK cells subpopulations, statistically significant differences emerged between the AML and control groups, with a lower number of immature NK cells (*p* = 0.01), of mature NK cells (*p* = 0.053) and a higher number of hypermature NK cells in the AML group (*p* = 0.01; Figure 3).

**Figure 4:**
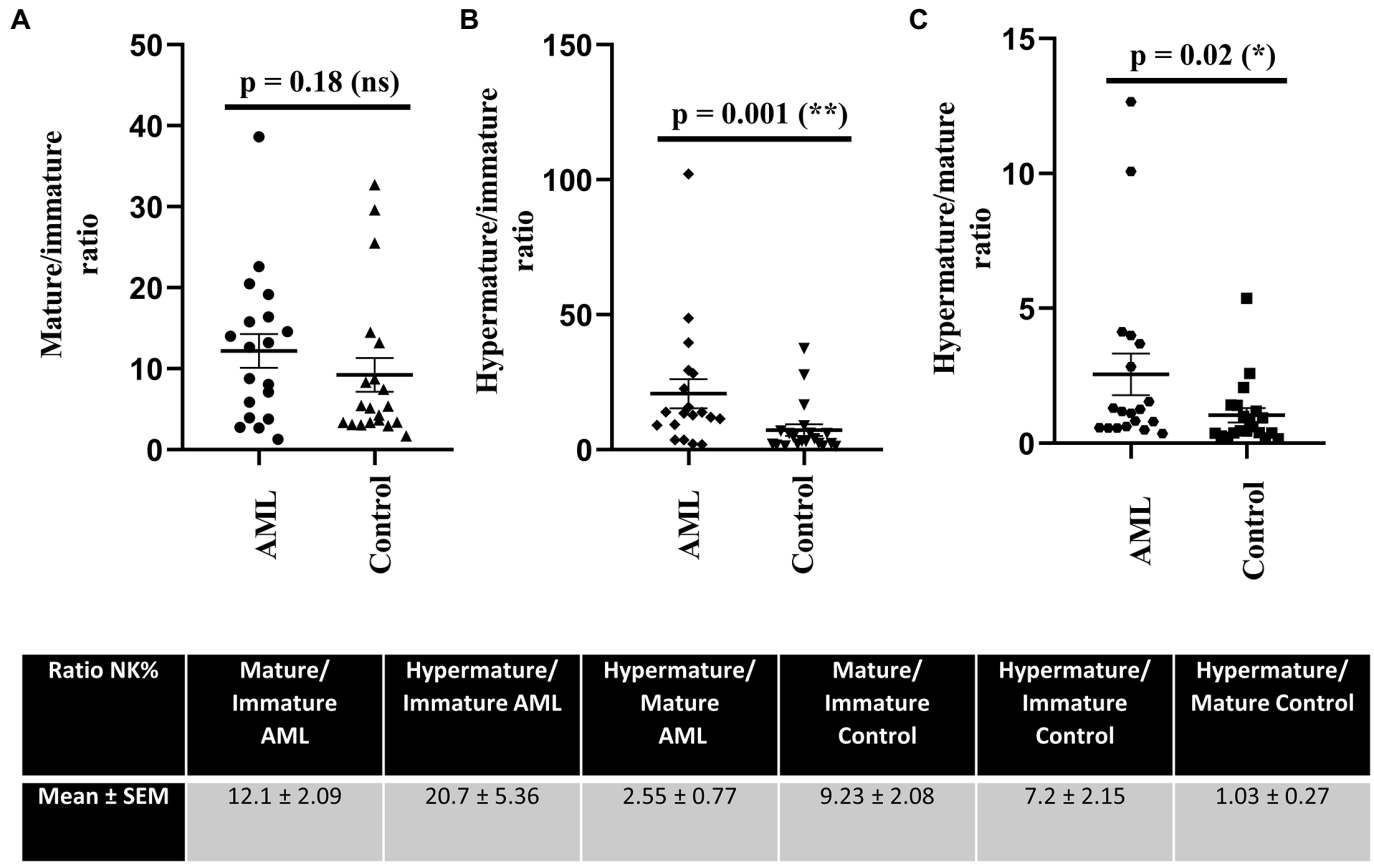
Ratios of NK cell percentages in each of the 3 maturation subsets for AML (*n* = 19) and healthy control (*n* = 20) cases. NK cells ratios for **(A)** mature to immature, **(B)** hypermature to immature, **(C)** hypermature to mature subsets in AML and healthy control individuals. Data is presented as scatter plots and the lines represent means ± SEM (*p* = 0.001 (**); p = 0.02 (*); ns - not significant; D’Agostino and Pearson normal distribution test was not passed; unpaired Mann–Whitney test). Table shows mean ± SEM of ratios between mature and immature subpopulations, hypermature and immature subpopulations and hypermature and mature subpopulations in the AML and healthy control groups.

Further, we have calculated the ratios of NK cells between the immature, mature and hypermature subgroups in order to assess if the lower number of immature NK cells and higher number of hypermature NK cells in the AML group are isolated features or characteristics of the same patient (Figure 4). All the mean ratios turned out to be higher in the AML group versus control, but only reaching statistical significance for the hypermature/immature NK cells in AML vs. control (20.7 ± 5.36 vs. 7.2 ± 2.15; *p* = 0.001) and the hypermature/mature NK cells in AML vs. control (2.55 ± 0.77 vs. 1.03 ± 0.27; *p* = 0.02). This is an important observation as it suggests that, despite a decreasing NK cell trend, the hypermature population is increased at the expense of mature and immature subsets in the same AML individual.

### 3.2. Abnormal distribution and expression of the nature killer inhibitory receptors within nature killer subpopulations in acute myeloid leukemia settings

**Figure 5:**
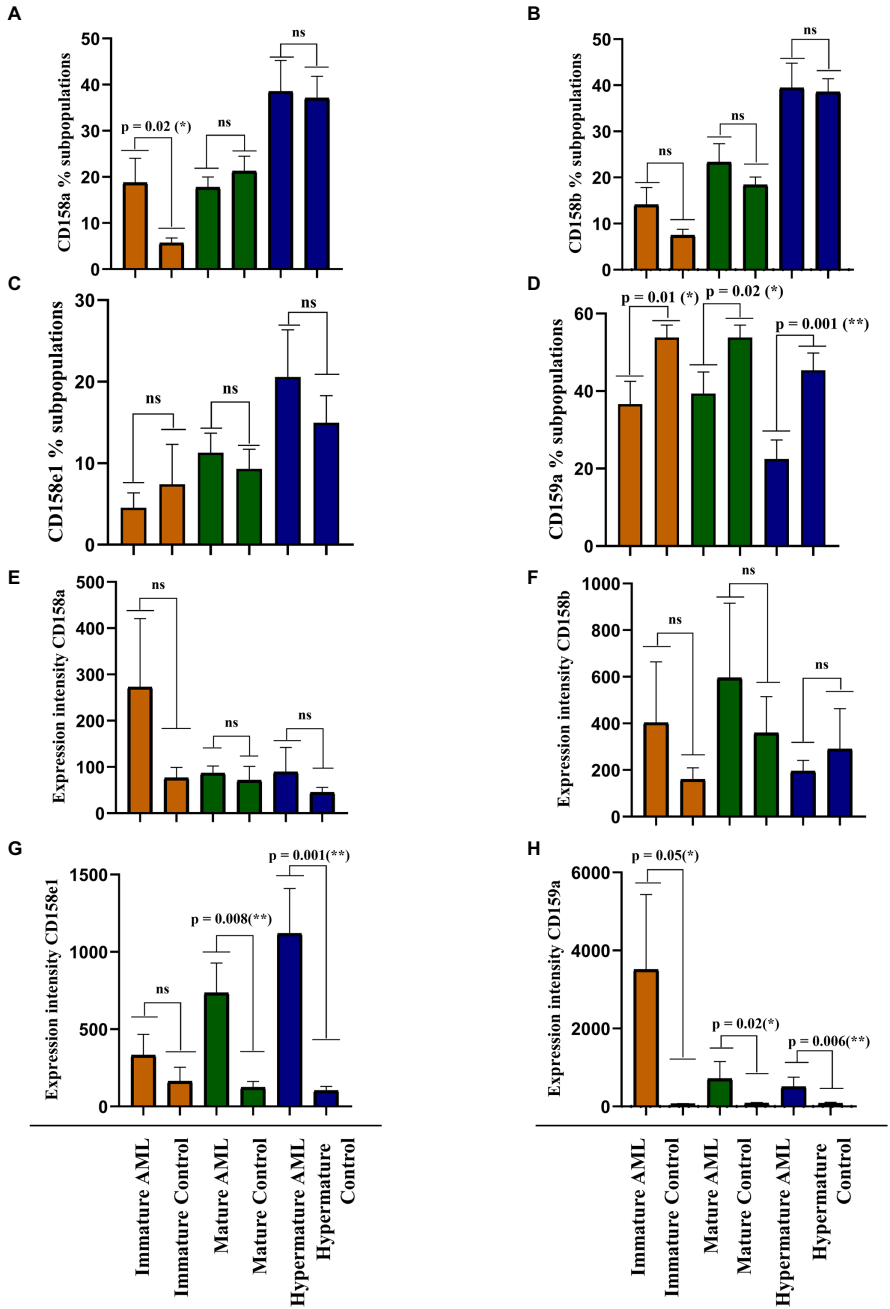
Comparison of cell percentages **(A–D)** and mean expression intensities **(E–H)** in distinct NK maturation subpopulations of AML (*n* = 19) and healthy control (*n* = 20) cases for each receptor. **(A,E)** CD158a; **(B,F)** CD158b; **(C,G)** CD158e1; **(D,H)** CD159a. Bars represent the mean ± SEM (*p* < 0.01 (**), p < 0.05 (*), ns, not significant; two tailed unpaired *t*-test or Mann Whitney test were applied for the data with or without Gaussian distribution, respectively).

Our next objective was to identify the differences of expression for the investigated NK inhibitory receptors within the 2 groups, across the 3 maturation stages described above (immature, mature and hypermature). Figures 5A–D presents the percentages of various NK cell subsets positive for the CD158a, CD158b, CD158e1 and CD159a inhibitory receptors, while Figures 5E–H presents the MFI index calculated for these four molecules.

A larger percentage of immature NK cells expressed CD158a and CD158b in AML than in the control group, but the difference was significant only for CD158a (*p* = 0.02; Figures 5A,B). Conversely, the percentages of CD158e1 and CD159a positive immature NK cells were higher in the control group than in AML, but only CD159a reached statistical significance (*p* = 0.01; Figures 5C,D). However, when analyzing the MFI index we could show that the expression level of each receptor was higher in the AML group, with a statistical difference for CD159a (*p* = 0.05; Figures 5E,F). We would also like to point out to the extremely high fluorescence values recorded when investigating this receptor, reflected by the high error bars generated by two outliers: a patient with complex karyotype and the patient with mutated FLT3.

The percentages of mature NK cells positive for the four receptors showed a different distribution. CD158b and CD158e1 tend to be expressed by more mature NK cells in AML than in the control group (Figures 5B,C), while CD158a and CD159a positive mature NK cells are better represented in the control group than in AML, with a statistical significant difference for CD159a (*p* = 0.002; Figures 5A,D). Again, the expression level of these receptors tended to be higher in the AML mature NK cells than in the control group, ranging from just a marginal difference for the expression of CD158b to statistical significance in the case of CD158e1 (*p* = 0.008) and CD159a (*p* = 0.02; Figures 5E–H).

Finally, the percentages of CD158a, CD158b, and CD158e1 were similar among the AML and control groups (Figures 5A–C), while the CD159a positive hypermature NK cells were clearly better represented in the control group (*p* = 0.001; Figure 5D). However, in terms of expression level, the situation was slightly different, with higher levels of CD158e1 (*p* = 0.008) and CD159a (*p* = 0.006) on hypermature NK cells in AML (Figures 5E,G,H), while the expression of CD158a and CD158b were not significantly changed (Figure 5F).

The expression intensity among the 2 groups and across the 3 subpopulations was extremely heterogenous for each receptor and several outliers could also be identified. Perhaps the most interesting outlier was present in the CD159a + immature AML subpopulation (Figure 5D), a case also associated with complex karyotype blast cells.

### 3.3. Killer immunoglobulin-like receptors and HLA-C genotypes evaluation in acute myeloid leukemia settings compared with healthy donors reveals differences in the inhibitory/activating killer immunoglobulin-like receptors receptor genes repertoire

**Figure 6:**
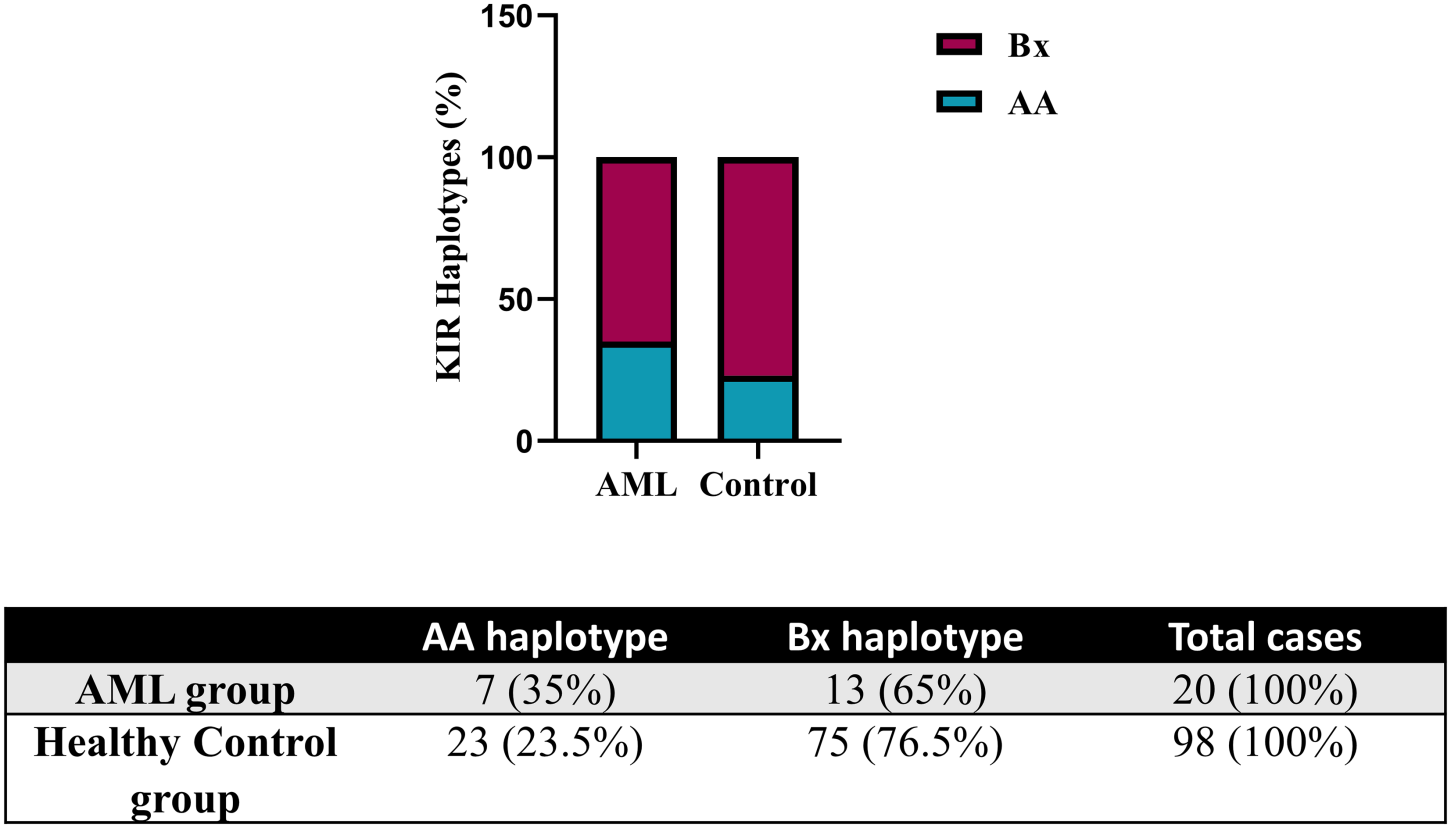
Frequency of KIR haplotypes in AML and healthy control individuals. Bars represent the percentages of AA (blue) and Bx (red) haplotypes of KIR genes (*n* = 20 for AML, *n* = 98 for the healthy control group).

Further, we evaluated the KIR and HLA-C genotypes and the results were compared against a control group of donor volunteers (*n* = 98) typed for both KIR and HLA-C genes. According to the KIR genotypes, the subjects of our study were distributed to the mostly inhibitory AA haplotype and the activating Bx haplotype, respectively. Data are summarized in Figure 6. The AA haplotypes seem to be better represented in the AML group than in the control one, but no statistical significance emerged (35% vs. 23.5%, *p* = 0.06; Figure 6).

**Figure 7:**
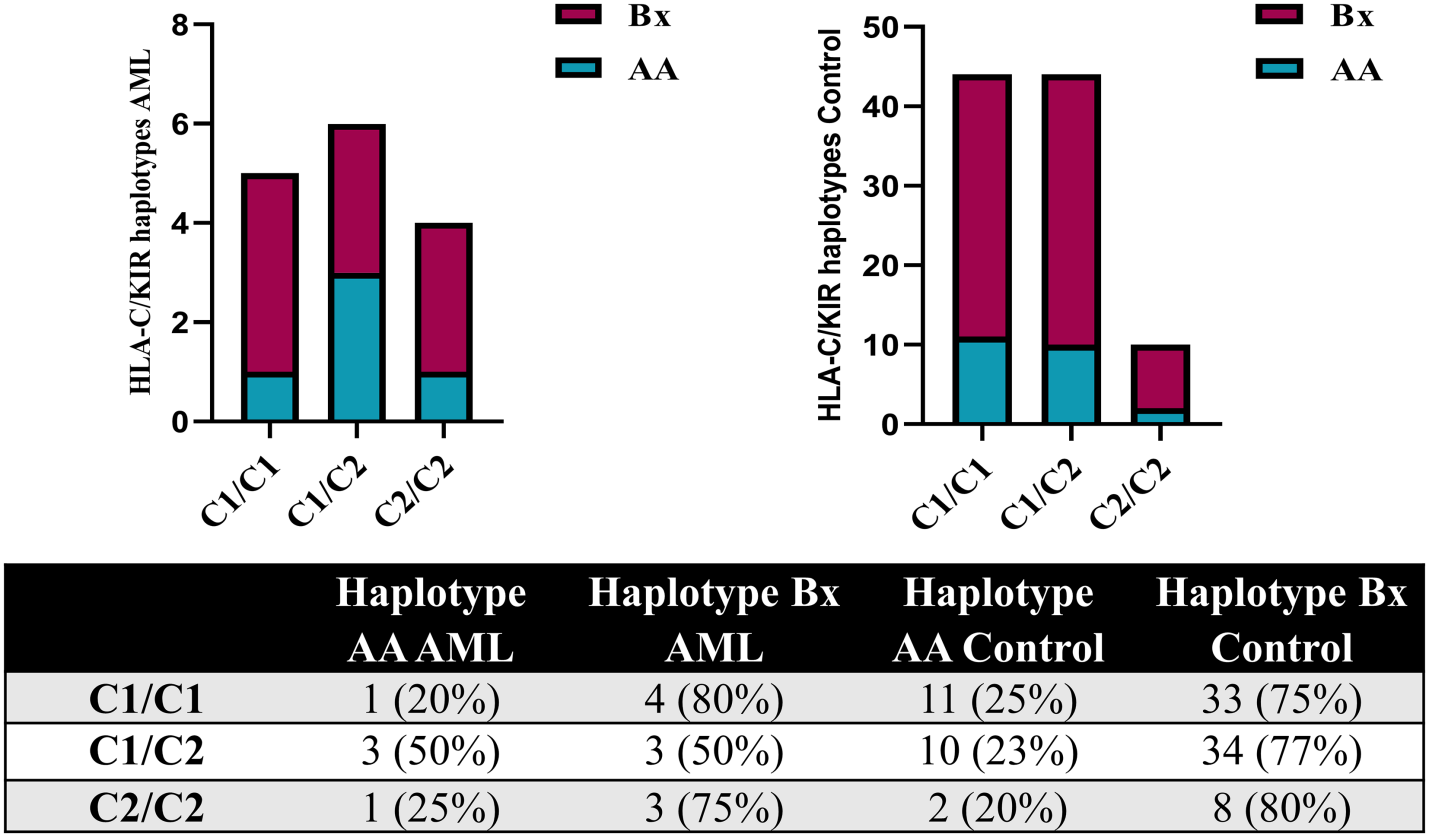
Combined HLA-C – KIR haplotypes in AML and healthy control individuals. Bars represent the number of (left) AML (*n* = 15) and (right) healthy control (*n* = 98) cases based on their HLA-C group (columns) and KIR haplotype (AA, blue; Bx, red).

The analysis of the HLA-C genotypes allowed us to distribute our subjects in the C1/C1, C1/C2 and C2/C2 groups and to match these groups with the AA and Bx KIR haplotypes. The Bx haplotype was best represented in all control groups, but in the C1/C2 AML group, the AA and Bx haplotypes were equally present (Figure 7).

**Figure 8:**
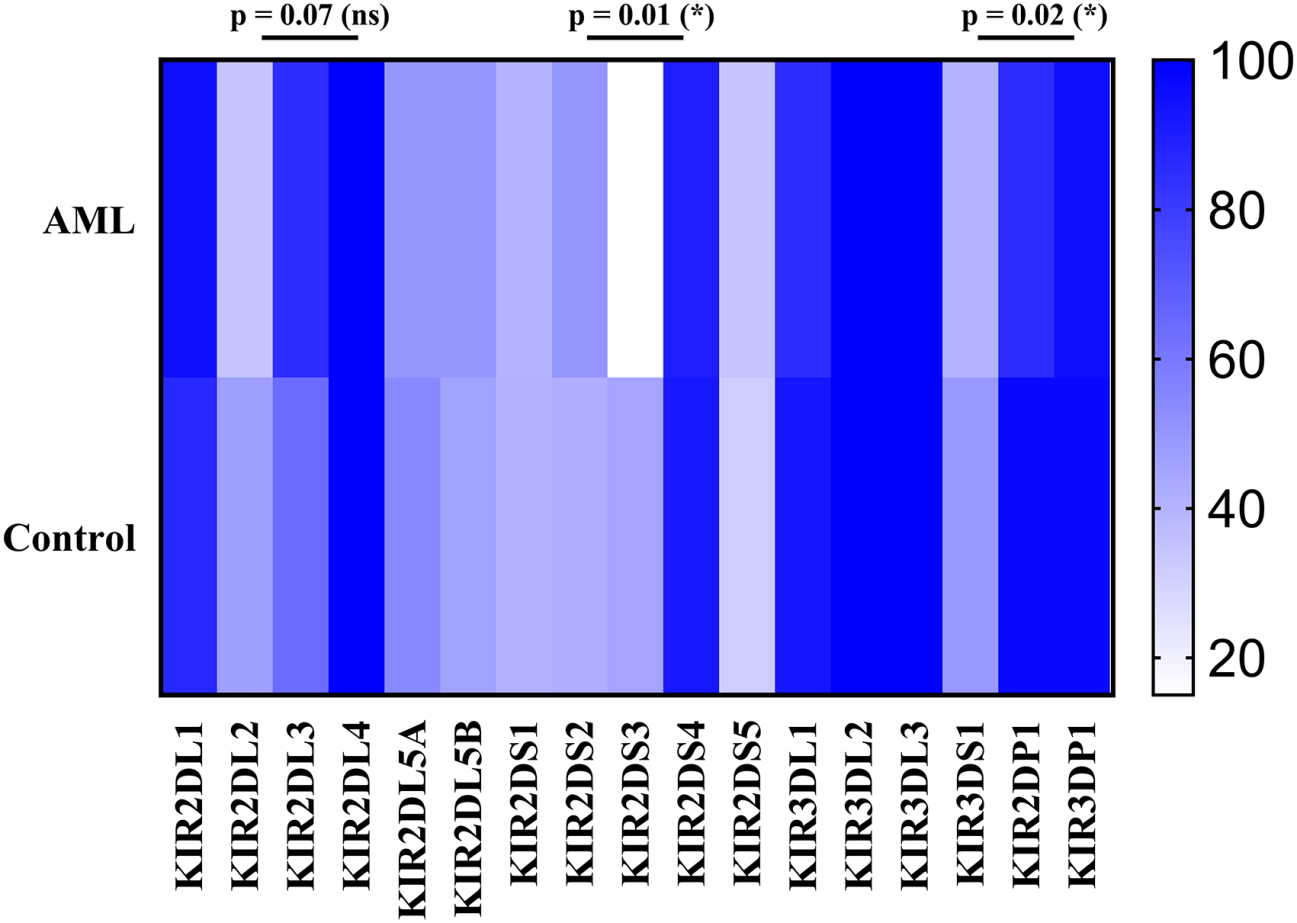
Heatmap of the individual KIR genes for the (top) AML and (bottom) Healthy Control groups. Color intensity ranges from white (mean < 20%) to dark blue (mean > 99%). Unpaired t-tests were used to compare the means of the analyzed genes between the 2 groups (*p* < 0.05 (*); ns, not significant).

We have further detailed the prevalence of each of the inhibitory, activating and pseudogenes composing the AA and Bx haplotypes in Table 3 and Figure 8. As expected, the framework genes KIR3DL2, KIR3DL3, KIR2DL4 and KIR3DP1 were present in more than 95% of the analyzed cases. No significant differences emerged when comparing the frequencies of the inhibitory KIR receptor genes of the AML and control subjects, with the exception of the 2DL3 gene, more frequent in the AML patients’ genomes. However, this difference was only marginal (85% vs. 64.3%; *p* = 0.07; Figure 8).

**Table 3:** The frequency of distinct KIR genes (%) in the AML and healthy control groups.

Inhibitory KIR	2DL1	2DL2	2DL3	2DL4	2DL5A	2DL5B	3LD1	3DL2	3DL3
AML (%)	95	35	85	100	50	50	85	100	100
Healthy group (%)	86.7	46.9	64.3	100	54.1	45.9	92.9	100	100
Activating KIR	2DS1	2DS2	2DS3	2DS4	2DS5	3DS1	2DP1	3DP1	
AML (%)	40	50	15	90	35	40	85	95	
Healthy control (%)	40.8	41.8	44.9	91.8	31.6	49	96.9	96.9	

Inhibitory KIR genes are presented in yellow, activating KIR genes in blue and pseudogenes in white.

As for the activating receptor genes, similar KIR gene frequencies could be noticed between the AML group and the control, except for the 2DS3 gene, that was significantly less frequent in the AML group compared to the control (15% vs. 44.9%; *p* = 0.01; Figure 8). Another significant difference is offered by the KIR2DP1 pseudogene, which was found in 85% of AML cases vs. 96.9% cases of the control group (*p* = 0.02; Figure 8).

From a general perspective, one may notice that the complex tumor karyotype was associated only with the C2 AML group (C1/C2 and C2/C2 genotypes) and mainly with a KIR AA genotype, while the normal tumor karyotype, seen in both C1 and C2 groups, was generally associated with a KIR Bx genotype. Interestingly, an increased heterogeneity in the percentage of NK cells expressing the four inhibitory receptors was detected in the C2 group compared to C1 individuals (Supplementary Figure S2).

## 4. Discussion

Since their discovery by Kärre et al. (7), the NK cells’ complexity was progressively unveiled over the years. Acknowledged initially just for their cytotoxic capabilities and their role in clearing tumor cells and virally infected cells without prior sensitization (41), they are now reconsidered in terms of ontogeny and instruction, activation and self-tolerance, as well as for their remarkable ability to secrete cytokines and thus regulate the activity of many other cells, including those of the immune system (42–44). Although they are similar to the cytotoxic T cells, they are differentiated by a fundamental characteristic: they possess an impressive repertoire of encoded germline receptors and have no possibility to rearrange receptor genes in an antigen specific manner (45). NK receptors fall into two major groups, activating and inhibitory, and the induction of a cytotoxic response plays upon a delicate balance between the two types of receptors, where the activating signals need to dominate in order to induce this effect (46, 47). However, if cytotoxicity is a feature predominantly associated with mature stages of differentiation, the immature NK cells are rather responsible for cytokine secretion (48).

In the present study we aimed to explore not only the NK maturation subpopulations and their expression of the four selected inhibitory receptors (3 iKIRs and NKG2A) in AML patients, but also to place these data in the context of the KIR genotypes and HLA-C profiles. KIR individual haplotypes present an impressive diversity due to allelic polymorphism and inheritance of different genes encoding activating and inhibitory receptors, which are known to bind HLA class I molecules (49). For instance, KIR2DL1 (CD158a) and KIR2DL2/2DL3 (CD158b) bind to HLA-C, while KIR3DL1 (CD158e1) binds to HLA-A and HLA-B (20). The absence of such molecules on the surface of a particular cell (generally described as “missing self”) (7) makes it a target for the NK attack. The HLA-C molecules are of particular interest as they can present two different binding sites for KIRs based on a single amino acid difference. As previously described, if an asparagine is present at position 80 in the α1 domain, these HLA-C molecules fall in the C1 group, and this particular epitope leads to a selective binding capacity to KIR2DL2 and KIR2DL3, while a lysine in the same position will characterize the C2 group and will offer binding selectivity to KIR2DL1 (49, 50). NKG2A (CD159a) pairs with CD94 to make an inhibitory receptor that binds to HLA-E, a non-classical MHC, which is not routinely investigated (21).

Unlike our previous study, where we evaluated functional NK cells from the BM in a normal setting and under the influence of a tumor microenvironment in myelodysplastic syndromes (MDS) and AML, here we have focused on PB NK cells. Based on gating parameters (FSC, SSC) and expression of specific surface markers (CD56, CD45, CD19, and CD3) we could identify NK cells and exclude B and T lymphocytes. We demonstrated that the percentage of NK cells was significantly lower in our AML group than in the control one, while practically no differences could be noticed for the B and T cells. Moreover, by targeting CD94, CD16 and CD57 molecules we were able to further discriminate the 3 main subpopulations: CD56 + CD94 + CD16-CD57- (immature); CD56 + CD94 + CD16 + CD57- (mature) and CD56 + CD94+/-CD16 + CD57+ (hypermature). We could thus show lower numbers of both immature and mature NK cells in AML, while the hypermature cells were significantly increased. Nonetheless, this increase alone was not able to compensate for the total NK percentage drop in AML. These results are in contradiction with our previous article where we showed the mature and hypermature NKs from the BM were quite similar in expression in AML and healthy control, while the immature NKs were more numerous in the AML group (*p* = 0.04) (38). However, in our current study addressing PB NK cells, we remarked that there is a significant enlargement of the hypermature compartment in the AML group compared to healthy controls, while the other subsets appeared to be decreased. This can be partially explained by the slightly larger group of AML patients included in the present study and, perhaps, by the differences between the bone marrow NK cells and those in the peripheral blood. Probably due to technical constraints, most studies addressing this topic focused on the PB and it is not entirely clear to what extent the periphery matches the distribution of NK subpopulations in the AML BM, a tumor microenvironment known for its ability to shape the T cell response (51, 52), and alter the NK development, with a loss of the immature subpopulation (53, 54) and compensatory increase in mature NK cells (55). On the other hand, other studies have shown that the mature compartment is rather diminished in myeloid malignancies (38, 56). Furthermore, given the ability of BM derived NK cell precursors to migrate into extramedullary sites, it is plausible that under such strenuous conditions NK cells might have the capacity to differentiate in the periphery, in lymphoid or non-lymphoid tissues, thus generating a distinctive immune landscape (57). Conversely, once matured, NK cells can traffic to various immune and non-immune tissues under the influence of various chemokines and exert their functions (58–61). The discrepancy between the drop in the total percentage of NK cells in our AML patients and the higher percentages of hypermature NK cells led us to evaluate the ratios between the NK subpopulations. We were thus able to show that both ratios between hypermature vs. immature and hypermature vs. mature NKs were statistically significant (*p* = 0.001 and *p* = 0.02, respectively). These data suggest that the differences in NK cell subpopulations between AML and control groups are not isolated events but are rather present in most AML patients as they might actually reflect a process in which the immune system is such modulated in order to push the NK differentiation and lead to the persistence of the highly cytotoxic CD57+ cells. This should be perceived as a desired type of differentiation, generating efficient effectors, able to kill cancer cells. However, the decrease of the total number of NK cells suggests that, overall, the immune system of the AML patients is hindered and unable to supply a sufficient number of such effectors.

Even though previous reports have demonstrated associations between leukemic blasts and NK cells (62), no correlations could be found in our study, perhaps due to the relatively small sample size. However, positive correlations between the T and B cell compartments and NK cells emerged. Different clinical studies demonstrated that in AML, the tumor-induced transcription factors dysregulation leads to a hindered proliferation of helper and cytotoxic T cells, faster T cell exhaustion and augmentation of the T cell regulatory compartment (63, 64). The T and the B cells should be thus further investigated in order to establish which subpopulations are actually expanded and what immune effector module is eventually favored, but this was beyond the aims of our study.

Instead, we expanded the investigation of the NK cells inhibitory receptors, both in terms of genotypes and surface expression. We have initially assessed the entire set of KIR genes by PCR and, by determining the genotype, we could further divide our subjects into the AA haplotypes including predominantly inhibitory KIRs and Bx haplotypes, which include mostly activating KIRs and are considered more advantageous for anti-tumor responses. In the setting of allogenic bone marrow transplant (alloHSCT), the consensus is that patients who are mismatched with their donors in the iKIR genes have better graft versus leukemia effects and lower risk of relapse (17, 65, 66). This may also suggest that patients with the AA haplotype may present a more ineffective NK mechanism of controlling tumorigenesis and therefore have a higher predisposition to developing AML. This theory was supported by Stringaris et al. (67) who demonstrated that patients with AA haplotype predicted a higher risk of transformation from MDS to AML and regarded AA haplotype as an independent risk factor. In both AML and control group subjects, the Bx haplotype was better represented than AA, with a Bx/AA ratio of 1.85 vs. 3.25. The AA haplotype proportion was higher in our AML group, indirectly reinforcing this hypothesis (35% vs. 23.5%, *p* = 0.06).

These data regarding the AA and Bx KIR haplotypes were further corroborated with the ones offered by the HLA-C genotyping, since these classical MHC molecules are main ligands for KIRs. We could thus show that while the C1/C2 AML patients were equally accompanied by AA and Bx haplotypes in terms of distribution, the control group showed an important bias toward the Bx haplotype (77% vs. 22%), unlike the C1/C1 and C2/C2 allelic groups where a similar large predominance of the Bx haplotype could be noticed for both the AML and control groups. Studies have shown that patients falling in the HLA-C1 (C1/C1) group were less susceptible to treatment related mortality than patients exhibiting HLA-C2 group (C1/C2, C2/C2) in hematopoietic stem cell transplant with self-matched HLA-C (68, 69). In this context, we could assume that HLA-C2 group, taken together with KIR AA haplotype, might explain a higher susceptibility to developing the disease or predict a poorer prognosis. In our study group, 67% of the AML patients had an HLA-C2 allotype, while only 54% of the normal subjects presented with it; however, this difference was not statistically significant.

The detailed KIR genotype analysis allowed us to calculate and compare the individual gene prevalence. Among the few differences between the AML and the control we noticed that the 2DL3 gene was found in 85% of the AML patients and in only 64.3% subjects of the control group (*p* = 0.07), while the 2DS3 gene was found in 15% of the AML patients, but in 44.9% within the control group (*p* = 0.001). This discrepancy might have a functional impact since KIR2DS3 is recognized as a protective factor against AML by Shahsavar et al. (70), to which should be added that some allelic variants that include also the common KIR2DS3*001 allele are heavily under expressed on the cell surface (71).

Given such potential differences between genotypes and phenotypes, we went further to investigate the surface expression of three inhibitory KIRs (KIR2DL1 (CD158a), KIR2DL2/2DL3 (CD158b), KIR3DL1 (CD158e1)) and NKG2A by flow cytometry. These receptors were considered for analysis due to their role in NK education, licensing or cytotoxic response limitation, reflected in the ability to modulate the immune response, hence the disease clinical course (72–75). The results of our investigation show an impressive heterogeneity in terms of NK cells percentages expressing these receptors, as well as the number of molecules expressed by these cells. CD158a is found on an increased percentage of immature NK cells in the AML group vs. control, and the level of expression is also increased. No major differences were noted for the mature and hypermature NK cells. CD158b is expressed by a larger percentage of immature and mature NK cells, accompanied by an increase of expression level in the AML group, while the hypermature cells tended to display a lower level of expression. CD158e1 is expressed by a lower percentage of immature cells, but with a higher level of expression; instead, more mature and hypermature cells present this receptor with a higher intensity. Finally, CD159a is constantly expressed by a lower percentage of immature, mature and hypermature NK cells in the AML group, but the level of expression is higher in all three subpopulations. One might speculate that the upregulation of these inhibitory receptors is tumor-induced, since it is known that iKIRs are strongly associated with NK licensing and ability to limit the immune response and CD94/NKG2A receptor delivers inhibitory signals in both NK cells and CD8+ T cells *via* interaction with its cognate ligand, HLA-E (76, 77).

Nonetheless, statistical significance was reached only for the increased percentage of CD158a positive immature cells, and decreased percentages of CD159a immature, mature and hypermature NK cells on one side, and for the increased levels of expression of CD158e1 and CD159a on mature and hypermature NK cells for the AML group on the other side.

Chretien et al. (78) were able to show that AML patients with a particular mutation (inversion 3) developed a CD16+ CD56- unconventional NK subpopulation, representing more than 20% of the total NK cells. Combined with the lower percentages of conventional NKs, this might offer the basis for an important immune escape mechanism.

In our previous paper (38) we have made an observation that one outlier stood out: an AML case with the FLT3 mutation which presented the highest percentage of total NK cells (55%) and, also, the highest proportion of CD159a + hypermature NK cells. In our current study, the only FLT3 case present was also an outlier, presenting an extremely low amount of NK cells (0.52%). Interestingly enough, the NK cells of this patient were the most numerous across all subsets of CD159a. A follow-up study addressing the potential role of this receptor in AML patients with FLT3 mutations could be worthy, especially in the era of emerging immunotherapy agents (79). Furthermore, this is strengthened by the variations in the expression levels of the investigated inhibitory receptors recorded in the present study and the remarkably high fluorescence intensities displayed by the NK cells of the patients with complex karyotypes.

There are many literature data available showing that tumor cells are able to modulate the expression of key surface proteins in order to evade immune responses, and this stands true for hematological malignancies, as well as for solid tumors (76, 80–82). In our study, we have shown that in AML, all in all, the inhibitory receptors displayed a general increased expression on all NK subpopulations. This increase represents another facet of the immune system’s impaired ability to adequately fight tumor cells that should be corroborated with the decrease in the total number of NK cells in AML. Furthermore, when analyzing NK cells subpopulations, we could notice that the percentage of hypermature cells was increased, a feature which suggests that somehow the NK cells are trying to compensate, to a certain extent, the fall in total number with a larger number of highly cytotoxic effectors. Anyhow, the flow cytometry analysis of the NK cells subpopulations and of the inhibitory receptors expression in AML is showing us not only a profoundly altered immune landscape, but also its diversity and heterogeneity.

## 5. Conclusion

In the present study, we analyzed the NK cells in AML patients in terms of numbers, distribution across maturation stages or inhibitory receptors expression and placed these pieces of information in the context of the genetic background offered by the KIR haplotypes and corresponding HLA-C ligands. A higher proportion of the mainly inhibitory KIR haplotype, AA, in the AML patients, along with a significantly lower KIR2DS3 in the patients exhibiting the Bx haplotype indicates to a potential genetic predisposition in the NK cells that allows for inefficient immune response and tumor escape in the setting of AML. In addition to this, the immunophenotyping showed that the AML patients are characterized by decreased percentages of PB NK cells, with lower immature and mature subpopulations, but a larger proportion of hypermature NKs. The investigation of a set of inhibitory receptors by flow cytometry revealed a very complex and diverse pattern of expression, but we could notice a general tendency for a higher expression level in all NK subpopulations. Nonetheless, AML patients with complex karyotypes or displaying a FLT3 gene mutation, besides falling exclusively in the C2 group, proved to be extreme outliers in terms of very low NK cells percentages or high inhibitory receptors expression.

Flow cytometry is thus of particular interest as it is able to offer intimate information which would allow us to better understand the personal profile of a particular patient and offer key data regarding receptors (some shared with the CD8+ T cells) targeted as check point inhibitors in immunotherapy, especially in individuals with complex tumoral karyotypes as it can potentially predict an erratic NK behavior and expression.

## Data availability statement

The original contributions presented in the study are included in the article/Supplementary material, further inquiries can be directed to the corresponding authors.

## Ethics statement

The studies involving human participants were reviewed and approved by Regional Institute of Oncology Ethics Committee. The patients/participants provided their written informed consent to participate in this study.

## Author contributions

VC, CR, MP-T, C-MA, and PC were involved in study design, data analysis, statistical analysis, manuscript drafting, and revision. VC, AD, and CD were involved in patient diagnosis, patient selection, and clinical investigation. VC, MP-T, and SH were involved in sample preparation, data acquisition, and analysis. All authors contributed to the article and approved the submitted version.

## Funding

This research was co-funded from the European Social Fund – the Human Capital Operational Program, Project Grant No: *POCU/993/6/13/154722* and the Operational Program for Competitiveness 2014–2020, Axis 1, under *POC/448/1/1* Research Infrastructure Projects for Public R&D Institutions/Universities “Multidisciplinary Medical Research-Development Platform in the NE region” CENEMED, mySMIS code: 127606.

## Conflict of interest

The authors declare that the research was conducted in the absence of any commercial or financial relationships that could be considered as potential conflict of interest.

## Publisher’s note

All claims expressed in this article are solely those of the authors and do not necessarily represent those of their affiliated organizations, or those of the publisher, the editors and the reviewers. Any product that may be evaluated in this article, or claim that may be made by its manufacturer, is not guaranteed or endorsed by the publisher.
