# Supplementary material for: Combined flow cytometry natural killer immunophenotyping and KIR/HLA-C genotyping reveal remarkable differences in acute myeloid leukemia patients, but suggest an overall impairment of the natural killer response
Source: Front Med (Lausanne). 2023 Mar 7;10:1148748. doi: 10.3389/fmed.2023.1148748 (PMC10028202; doi:10.3389/fmed.2023.1148748)
Supplement: Supplementary file 1 [file Data_Sheet_1.docx]

Supplementary Material

**Combined flow cytometry NK immunophenotyping and KIR/HLA-C genotyping reveal remarkable differences in AML patients, but suggest an overall impairment of the NK response**

Vlad Andrei Cianga^1,2^, Cristina Rusu^3*^, Mariana Pavel-Tanasa^4^, Angela Dascalescu^1,2^, Catalin Danaila^1,2^, Sebastian Harnau^4^, Carmen Aanei^5,6^, Petru Cianga^4*^

1. Department of Hematology, University of Medicine and Pharmacy “Grigore T. Popa” Iasi, Romania
2. Department of Clinical Hematology. Regional Institute of Oncology, Iasi, Romania
3. Department of Genetics, University of Medicine and Pharmacy “Grigore T. Popa” Iasi
4. Department of Immunology, University of Medicine and Pharmacy “Grigore T. Popa” Iasi
5. Laboratory of Hematology, Nord Hospital, CHU Saint Etienne, Cedex2, France
6. INSERM U1059-SAINBIOSE, Université de Lyon, Saint-Etienne, France

*Correspondence

Cristina Rusu,

Petru Cianga, [petru.cianga@umfiasi.ro](mailto:petru.cianga@umfiasi.ro)

## Supplementary Figures


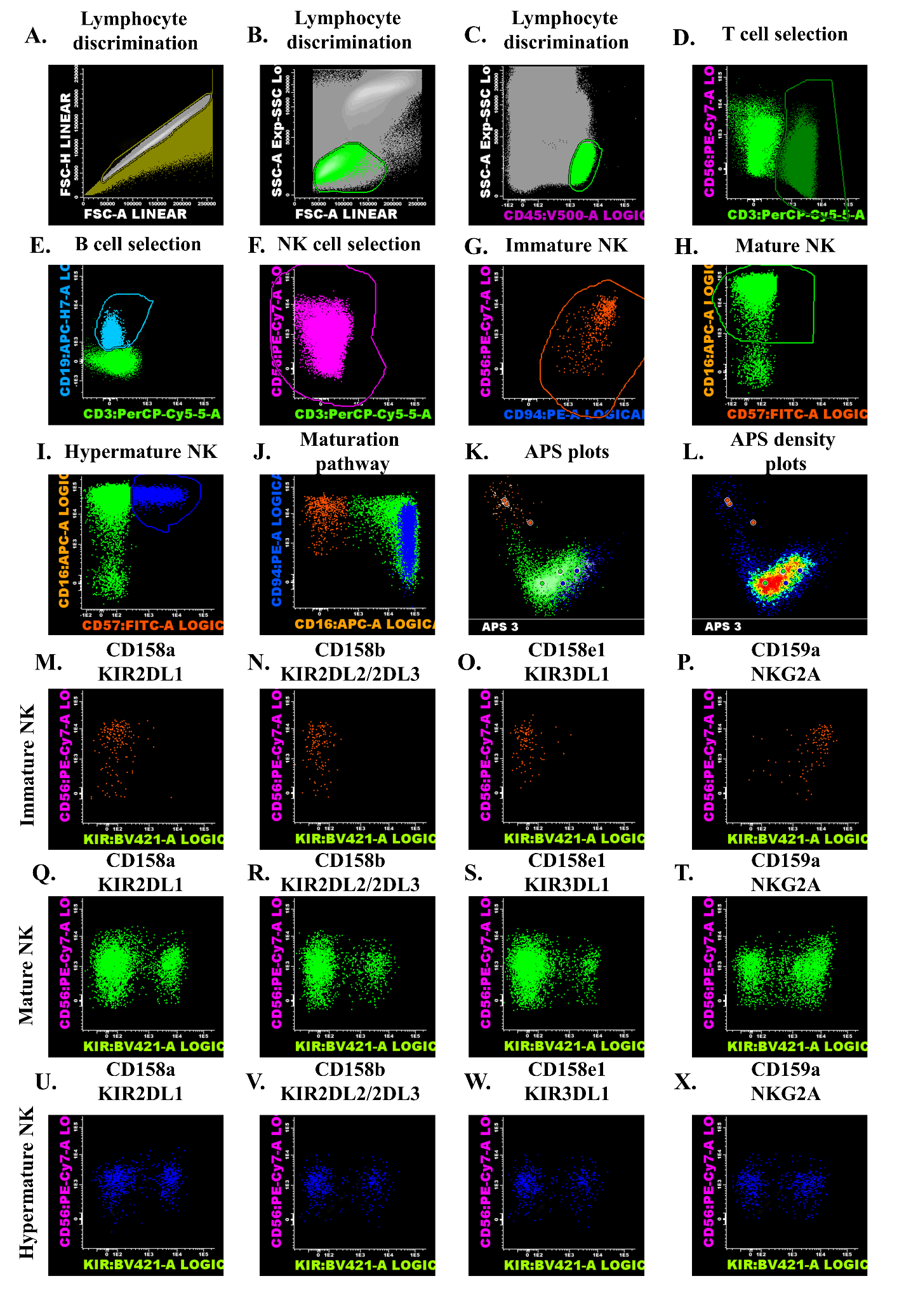


**Supplementary Figure 1.** **Example of gating strategy for NK cells and classification into 3 subpopulations:** CD56^bright^CD94+CD16-CD57- represents the immature subpopulation (orange dots); CD56^dim^CD94+CD16+CD57- represents the mature subpopulation (green dots); CD56^dim^CD94+CD16+CD57+ represents the hypermature subpopulation (blue dots); (A) Singlets were selected on the FSC-H and FSC-A plots. (C) The lymphocyte population was discriminated in the side scatter (SSC) *vs* CD45 plots. (D, E) B and T cells were excluded based on their expression of CD19 and CD3, respectively. (F) NK cells were evidenced as CD56+CD3-CD19- cells. Separation into immature (G; orange), mature (H; green) and hypermature (I, blue) subsets was realized based on expression of CD94, CD16 and CD57. The maturation pathway is shown in figure J. The automatic population separator (APS) which is based on the Principal Component Analysis (PCA) was used as a control for manual gating in all cases (K, L). Next, we evaluated the expression of CD158a, CD158b, CD158e1 and CD159 in the immature (M, N, O, P), mature (Q, R, S, T), and hypermature (U, V, W, X) subpopulations.


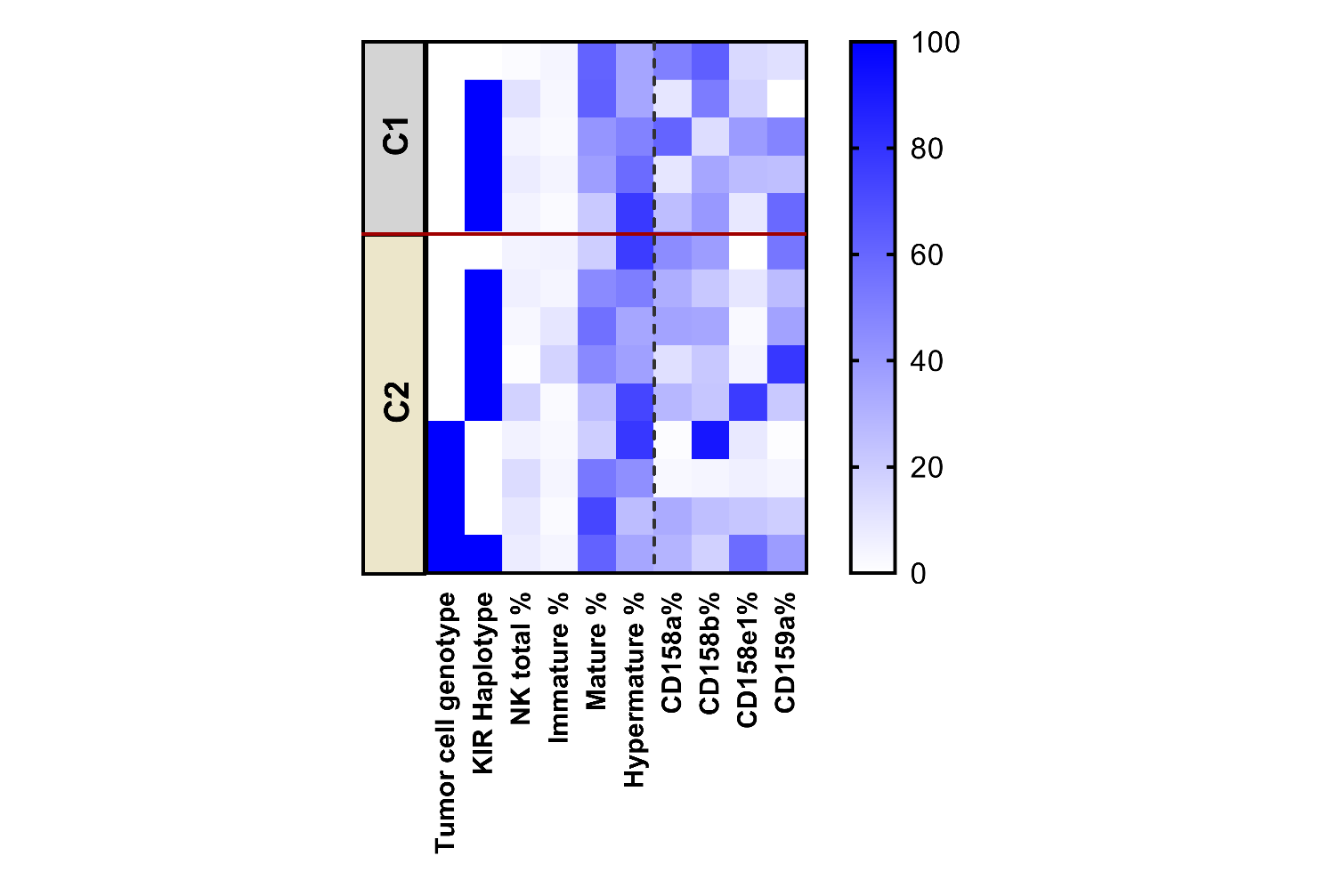


**Supplementary Figure 2.** **Heatmap showing various parameters in AML patients (columns).** First column shows tumor cell genotypes as being either normal karyotype (46XY/46XX) (white) or complex karyotype (blue); second column shows KIR haplotype as AA (white) or Bx (blue); Columns 3 – 8 show range of percentage (%) for total NK cells, immature, mature, hypermature and NK cells percentages (%) positive for CD158a, CD158b, CD158e1 and CD159a. Color intensity ranges from white (mean 0 %) to dark blue (mean 100 %).

1. **Supplementary Tables**

| **Antibody** | **Fluorochrome** | **Clone** | **Specificity** |
| --- | --- | --- | --- |
| **Anti CD57** | **FITC** | **HNK-1** | **Mouse anti human** |
| **Anti CD3** | **PerCPCy5.5** | **SK-7** | **Mouse anti human** |
| **Anti CD16** | **APC** | **3G8** | **Mouse anti human** |
| **Anti CD56** | **PeCy7** | **B159** | **Mouse anti human** |
| **Anti CD94** | **PE** | **HP-3D9** | **Mouse anti human** |
| **Anti CD19** | **APC-H7** | **SJ25C1** | **Mouse anti human** |
| **Anti CD45** | **V500** | **HI30** | **Mouse anti human** |
| **Anti CD158a** | **BV421** | **HP-3E4** | **Mouse anti human** |
| **Anti CD158b** | **BV421** | **DX27** | **Mouse anti human** |
| **Anti CD158e1** | **BV421** | **DX9** | **Mouse anti human** |
| **Anti CD159a** | **BV421** | **131411** | **Mouse anti human** |

1. **Supplementary Table. Antibody characteristics**
